# Community-Based Chronic Disease Prevention and Management for Aboriginal People in New South Wales, Australia: Mixed Methods Evaluation of the 1 Deadly Step Program 

**DOI:** 10.2196/14259

**Published:** 2019-10-21

**Authors:** David Peiris, Lachlan Wright, Madeline News, Katherine Corcoran

**Affiliations:** 1 The George Institute for Global Health, UNSW Sydney Newtown Australia

**Keywords:** chronic disease, screening, indigenous health, prevention, primary health care

## Abstract

**Background:**

Chronic diseases account for over 70% of health gaps between Aboriginal people and the rest of the Australian population. The 1 Deadly Step program involves community-based events that use a sporting platform and cultural ambassadors to improve chronic disease prevention and management in New South Wales (NSW).

**Objective:**

This study aimed to evaluate the feasibility and acceptability of a community-based chronic disease screening program for Aboriginal people.

**Methods:**

In 2015, the program was enhanced to include an iPad app for screening assessments, a results portal for nominated care providers, and a reporting portal for program administrators and implemented in 9 NSW community events. A mixed methods evaluation comprising survey data, analytics obtained from iPad and Web portal usage, and key informant interviews was conducted.

**Results:**

Overall, 1046 people were screened between April 2015 and April 2016 (mean age 40.3 years, 640 (61.19%) female, 957 (91.49%) Aboriginal or Torres Strait Islander). High chronic disease rates were observed (231 [22.08%] participants at high cardiovascular disease (CVD) risk, 173 [16.54%] with diabetes, and 181 [17.30%] with albuminuria). A minority at high risk of CVD (99/231 [42.9%]) and with diabetes (73/173 [42.2%]) were meeting guideline-recommended management goals. Overall, 297 participants completed surveys (response rate 37.4%) with 85.1% reporting satisfaction with event organization and information gained and 6.1% experiencing problems with certain screening activities. Furthermore, 21 interviews were conducted. A strong local working group and processes that harnessed community social networks were key to implementation success. Although software enhancements facilitated screening and data management, some technical difficulties (eg, time delays in processing blood test results) impeded smooth processing of information. Only 51.43% of participants had a medical review recorded postevent with wide intersite variability (10.5%-85.6%). Factors associated with successful follow-up included clinic managers with overall program responsibility and availability of medical staff for immediate discussion of results on event day. The program was considered highly resource intensive to implement and support from a central coordinating body and integration with existing operational processes was essential.

**Conclusions:**

1 Deadly Step offers an effective and acceptable strategy to engage Aboriginal communities in chronic disease screening. High rates of risk factors and management gaps were encountered, including people with no previous knowledge of these issues. Strategies to improve linkage to primary care could enhance the program’s impact on reducing chronic disease burden.

## Introduction

Chronic diseases, including cardiovascular disease (CVD), diabetes, chronic kidney disease (CKD), chronic respiratory disease and cancer, account for over 70% of health gaps between Aboriginal people and the rest of the Australian population [1]. Aboriginal and Torres Strait Islander peoples experience around 5 times greater CVD burden than other Australians [1]. Aboriginal people also experience substantial inequities in access to primary health care, and innovative, culturally safe strategies to improve access to high-quality chronic disease care and prevention are needed [2-5]. Studies of CVD risk management in Australian general practice and Aboriginal Community Controlled Health Service (ACCHS) settings demonstrated that 50% of routinely attending adults lacked sufficient recorded information to comprehensively evaluate vascular risk [6]. For those identified at high vascular risk, only around 40% were prescribed guideline-indicated medicines.

Community-based strategies that improve the uptake of best practice recommendations could both substantially reduce the disease burden from chronic diseases and help improve health system efficiencies. The 1 Deadly Step program was developed in partnership with New South Wales (NSW) Health and the Australian Rugby League to address the high prevalence of chronic diseases in NSW Aboriginal communities. The term *deadly* is used by many Aboriginal people to mean *awesome, great, excellent* and the term *1 Deadly Step* makes a link between rugby league and making a step toward good health. First implemented in 2012, it uses a culturally safe, innovative, community-based model in which annual events are held to increase awareness of chronic diseases and to promote prevention, early detection, and evidence-based management of chronic diseases through timely referral and follow-up. At each community event, consenting participants are taken through specific stations to assess chronic disease risk factors. Drawing on the popularity of rugby league in Aboriginal communities, the program uses this sporting platform to encourage local communities to participate. High-profile Aboriginal rugby league players from the local community are engaged as cultural ambassadors and are available on the event day to promote the importance of looking after one’s health.

In this paper, we describe the development of an electronic platform to support implementation of the 1 Deadly Step program and outline the findings from a mixed methods evaluation. It draws on the key findings from the full evaluation report prepared for the commissioning agency [7]. The objectives of this study were to (1) describe the demographic and chronic disease risk factor profile of program participants, (2) assess evidence-practice gaps for chronic disease management, and (3) assess program acceptability to both participants and providers and identify implementation barriers and enablers.

## Methods

### Program Enhancements

An earlier evaluation of the program in 2012 concluded that 1 Deadly Step events represented a successful community screening approach with high acceptability by the participating communities [8]. However, a key recommendation was the need for improvements to screening and data collection processes and support for systematic follow-up care. To help address these issues, an electronic platform was developed comprising 4 components.

#### iPad Screening App

A screening algorithm was developed as an iPad app. ACCHS staff were engaged to inform the design via a series of workshops and iterative testing of software prototypes. A local Aboriginal artist also provided the software design to improve the visual appeal of the app. The core elements of the system built are shown in Multimedia Appendix 1. It comprises a step-by-step screening process, a printable summary report and a real-time, secure upload of data to a standards-compliant repository.

#### Administrator Portal

A Web-based portal allows program staff to manage events (Multimedia Appendix 2). This included setting up test events for staff training purposes, registration of staff users responsible for entering data into the iPad on event days, and registration of nominated care providers who would be given access to the provider portal.

#### Provider Portal

For consenting participants, a clinical summary report is accessible from a password protected, secure data repository. The ACCHS or general practice can view data for the participants who have nominated them as their care provider (Multimedia Appendix 3). Up to 3 care providers can be assigned to each participant (eg, general practitioner [GP], ACCHS manager, and local health district [LHD] staff). The nominated care provider can generate a summary of the screening data and upload this document to the patient’s electronic record. The portal has a sort function that prioritizes patients according to their chronic disease risk and follow-up status.

#### Web-Based Reporting Tool and Site Evaluation Report

Program administrators can access aggregated data reports for each event, which includes the demographic and health profile of participants, numbers of assessments completed, and the nominated care providers. A site evaluation report was also provided by the research team postevent for dissemination to participating health care providers.

### Evaluation

The enhanced program was implemented by the NSW Agency for Clinical Innovation in 2015-2016. A program logic model (Multimedia Appendix 4) and evaluation framework using the Reach, Effectiveness, Adoption, Implementation, Maintenance framework was developed by the research evaluation team [9]. This involved detailed discussion with key stakeholders to identify core evaluation objectives and to design strategies and questions that appropriately measured those objectives.

**Table 1:** Data elements for evaluation of the 1 Deadly Step program.

Data element	Means of collection	Information collected
Screening assessment deidentified data	Secure access to the data repository	Demographic information; clinical information including cardiovascular disease risk, diabetes risk, kidney disease risk, and current treatment being received
Satisfaction surveys for participants	Anonymous paper survey at the end of a screening event	Satisfaction, acceptability, and utility of the program
Reporting website follow-up data	Deidentified data extract at end of project	Follow-up status of all participants screened
Key stakeholder interviews	Semistructured interviews with health service managers, local health district staff, clinical staff, and program staff	Satisfaction, acceptability, and utility of the program

A mixed methods approach was taken to data collection, and 4 main data sources were used to inform the evaluation (Table 1). Deidentified quantitative and identified qualitative data were used concurrently to gain a detailed understanding of the activities, inputs, and outputs of the project as identified in the program logic model.

#### Risk Factor Analyses

An assessment of the proportion of participants at risk of diabetes, CVD, and CKD was made. CVD risk estimation for those aged 30 years and older was based on the Framingham risk equation and National Vascular Disease Prevention Alliance guidelines [10]. High CVD risk was defined as any of the following: (1) a calculated 5-year CVD risk exceeding 15%, (2) presence of clinically high-risk conditions (including diabetes and age >60 years, diabetes and albuminuria, systolic blood pressure>180 mmHg, diastolic blood pressure >110 mmHg, or total cholesterol >7.5 mmol/L), and (3) a self-reported CVD diagnosis (coronary heart disease, cerebrovascular disease, and peripheral vascular disease). Diabetes risk was based on the Australian type 2 diabetes risk (AUSDRISK) screening tool [11], glycated hemoglobin (HbA_1c_), and the capillary blood glucose level taken on the event day. CKD risk was defined as any of the following: body mass index (BMI) greater than 30 kg/m^2^, current smoker, the presence of CVD, family history of CKD, and the presence of diabetes [12].

#### Care Practices

The proportion of participants identified with or at high risk of these conditions, who were accessing appropriate management (eg, self-reported use of guideline-recommended medications and attainment of recommended treatment targets) was assessed. Medication use was based on self-reporting. An information pop-up box was available in the iPad app with common medication names to assist in answering these questions.

#### Participant Satisfaction

At the end of their screening assessment, participants were asked to complete a 2 min survey seeking feedback on the overall event and any problems encountered at each of the screening stations.

#### Follow-Up

The nominated provider or manager was encouraged to record in the provider portal which participants were followed up and the date of follow-up. Data were extracted from the portal to assess follow-up rates at the end of the program (August 31, 2016).

For the quantitative data, simple frequency analyses were conducted using SAS software version 9.4 (SAS Institute) including assessing for variation by site, gender, and clinical characteristics.

#### Interviews

Semistructured interviews were conducted with a purposive sample of health service and program staff. A maximum-diversity sampling strategy was taken in which participants were selected on the basis of site, staff role, and involvement in the program. Interviews were generally conducted by telephone, with two evaluation team members taking an insider-outsider approach [13]—one (KC) who was not involved in the program design and the other (LW) an Aboriginal researcher with detailed program knowledge and a long history of engagement with the participating communities. Interviews were digitally recorded, professionally transcribed, and reviewed by a member of the evaluation team to ensure accuracy of the transcription. Thematic analysis was conducted, and themes were aligned with the areas of focus in the logic model. All team members met regularly to develop the coding framework and discuss the significance of the emerging codes and their relevance to the quantitative data that was being concurrently collected. This framework was iteratively revised, and member checking was informally conducted to ensure consistency of interpretation. Future interviews were modified to enable deeper exploration of particular emergent themes.

The evaluation was approved by the Aboriginal Health & Medical Research Council Human Research Ethics Committee. Formal approvals from each of the participating sites were obtained. Informed consent was obtained from all participants who were interviewed.

## Results

### Participant Characteristics

A total of 1046 people were screened between April 2015 and April 2016 at 9 events in NSW. Table 2 highlights the participant characteristics by site. An average of 116 participants was screened per site, with a larger proportion of females than males screened at all sites (61.2% vs 38.8% overall). The majority of participants (91.5%) identified as Aboriginal or Torres Strait Islander. The mean age of the participants was 40.3 years (range 15-79 years). On the basis of 2016 Indigenous area census data, 5.58% of the population older than 15 years was screened overall (range 3.0%-19.8%).

Data on chronic disease risk factors by gender are summarized in Table 3. For weight-related measures, 50.3% of the sample had a BMI in the obesity range (mean BMI 31.1 kg/m^2^) and 65.6% had an elevated waist circumference (>102 cm for men and 88 cm for women and BMI >40 kg/m^2^). There were significant gender differences with females recording higher rates of obesity (53.8% vs 44.8%) and elevated waist circumference (76.1% vs 49.0%). For smoking status, 37.2% were current smokers, with a further 6.7% having recently given up smoking in the previous 12 months. Most current smokers had been smoking for more than 10 years (64.1%) and 44.0% smoked more than 10 cigarettes per day. Smoking rates by gender were similar (34.7% males, 38.8% females). Importantly, 20.0% of those under 18 years reported being current smokers.

Data on the CVD risk of participants are summarized in Table 4. Around 1 in 5 (22.08%) of the sample was at high CVD risk, either through having an existing CVD condition or one or more clinically high-risk conditions. There were minimal differences in CVD risk profile by gender. Overall, 16.54% of the participants reported having diabetes (Table 5). An additional 4.30% of the sample had diabetic-range HbA_1c_ levels greater than or equal to 6.5% (48 mmol/mol) without a previous known diagnosis of diabetes. Another 28.87% of participants had potentially elevated glucose levels (random capillary blood glucose levels between 5.5 mmol/L and 11.1 mmol/L).

There were few gender differences in the diabetes risk profile of the sample. In total, 82.98% of participants were at high CKD risk and 17.30% had albuminuria (≥2.5 mg/mmol for males and ≥3.5 mg/mmol for females). There were negligible gender differences in the proportion of people at high risk of CKD overall.

**Table 2:** Demographic profile of participants screened.

Event site	Date	Number screened	Average age (years)	Female, n (%)	Aboriginal and/or Torres Strait Islander, n (%)	Local Aboriginal and/or Torres Strait Islander community^a^, %
1	04/17/2015	132	42.2	85 (64.4)	111 (84.1)	3.6
2	07/06/2015	114	40.6	71 (62.3)	113 (99.1)	3.0
3	10/26/2015	107	34.6	63 (58.9)	90 (84.1)	6.7
4	12/01/2015	118	41.6	71 (60.2)	114 (96.6)	4.5
5	03/06/2016	77	39.0	55 (71.4)	68 (88.3)	3.6
6	03/12/2016	119	38.4	60 (50.4)	114 (95.8)	8.9
7	03/17/2016	123	47.0	73 (59.4)	107 (87.0)	7.0
8	03/23/2016	127	40.0	81 (63.8)	119 (93.7)	11.2
9	04/06/2016	129	37.9	81 (62.8)	119 (93.8)	19.8
Total	—^b^	1046	40.3	61.19	957 (91.49)	5.58

^a^On the basis of 2016 Indigenous area census data for people aged 15 years and older.

^b^Not applicable.

**Table 3:** Chronic disease risk factors by gender

Risk factors	Female (N=640), n (%)	Male (N=406), n (%)
Current smoker	248 (38.8)	141 (34.7)
Body Mass Index >30 kg/m2	344 (53.8)	182 (44.8)
Elevated waist circumference (>102cm for males, >88cm for females)	487 (76.1)	199 (49.0)
Physical activity less than 2.5 hours/week	139 (21.7)	48 (11.8)
Infrequent fruit intake	185 (28.9)	100 (24.6)
Infrequent vegetable intake	526 (82.2)	337 (83.0)
Blood pressure >140/90 mmHg	200 (31.3)	185 (45.6)
Dyslipidaemia (Total cholesterol > 5.5, HDL <1, LDL >3.5, Triglycerides >2.0)	464 (72.5)	308 (75.9)

**Table 4:** Cardiovascular and diabetes risk profile for 1046 people.

Cardiovascular disease risk profile	n (%)
Low risk (<10% 5-year risk)	429 (41.01)
Medium risk (10-15% 5-year risk)	17 (1.63)
High risk (>15% 5-year risk)	15 (1.43)
Clinically high-risk condition present	79 (7.55)
Established cardiovascular disease	137 (13.10)
<30-year olds	312 (29.83)
Missing data	57 (5.45)

^a^Australian type 2 diabetes risk screening assessment.

**Table 5:** Diabetes risk profile for 1046 people.

Diabetes risk profile	n (%)
Low risk (AUSDRISK^a^ ≤6)	31 (2.96)
Medium risk (AUSDRISK 6-11)	145 (13.86)
High risk (AUSDRISK ≥12)	350 (33.46)
Impaired glycemia	302 (28.87)
Possible new diabetes diagnosis	45 (4.30)
Established diabetes	173 (16.54)

^a^Australian type 2 diabetes risk screening assessment.

**Figure 1:**
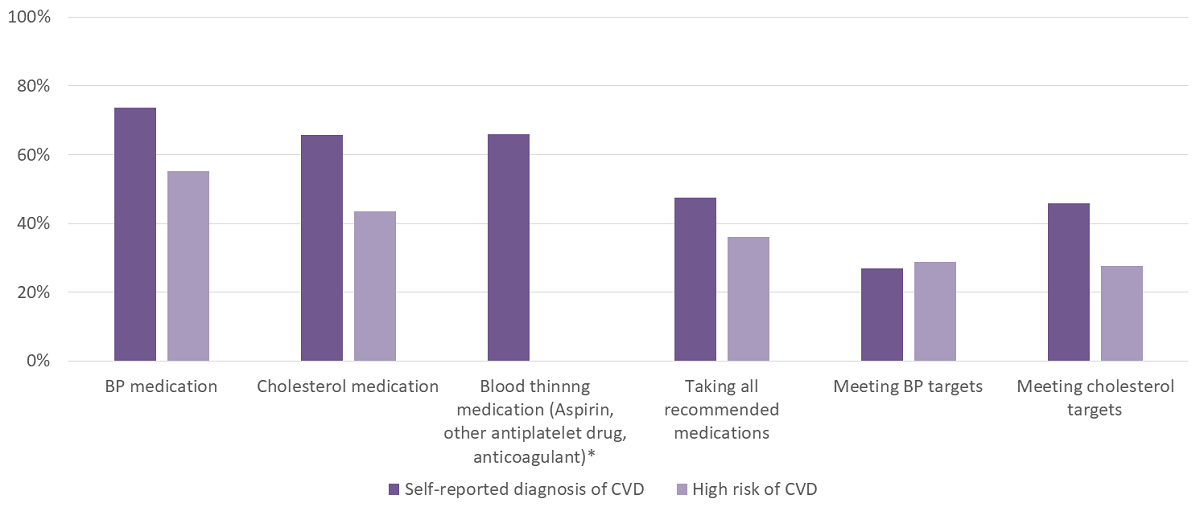
Management for people with or at high risk of CVD (n=231). BP: blood pressure; CVD: cardiovascular disease.

Figure 1 illustrates the proportion of patients with CVD and at high risk of CVD meeting various care practice parameters. Overall, only 42.9% of people with or at high risk of CVD were taking guideline-recommended treatments. There were no significant gender differences in those who reported currently taking these medicines.

For those with a known diagnosis of diabetes (n=173), the majority (80.4%) reported taking an oral glucose-lowering medication and 34.7% reported taking insulin. Overall, 42.2% were attaining a target HbA_1c_ of 7% or less (53 mmol/mol) and 61.9% were attaining a target HbA_1c_ of 8% or less (64 mmol/mol). A higher proportion of women met the HbA_1c_ targets than men (46.3% vs 35.4%, respectively).

As of August 2016, 538 of the 1046 participants screened had been recorded as having been followed up (51.43%). There was wide variability in the recording of follow-up rates (10.5%-85.6%). Participants with or identified to be at high risk of diabetes, CVD, or CKD had slightly higher follow-up rates than the total population at each site and overall.

### Satisfaction

**Figure 2:**
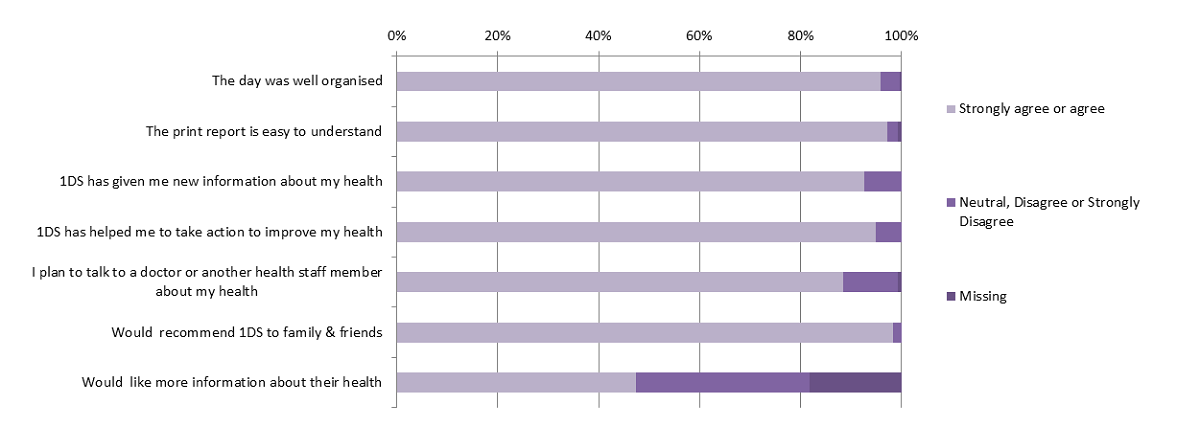
Participant survey of overall program impression (n=297). 1 DS: 1 Deadly Step.

A total of 297 participants completed satisfaction surveys at 7 events (response rate 37.4%). The overall impressions of the program were positive, with the vast majority of participants satisfied with event organization and the information gained from the event day (Figure 2). Similarly, the majority of responders encountered few problems when asked about specific screening stations, although around 6% of participants did report problems with the urine and blood testing stations. Free-text entries were also analyzed and were concordant with these findings.

### Interviews

A total of 21 interviews were conducted (Table 6). Interview themes have been organized to align with the three key stages of the program (pre-event, event, and postevent).

#### Pre-Event

#### Importance of the Working Group

An important initial stage in organizing an event involved the coordinating agency engaging local stakeholders to determine interest and capacity. A stakeholder working group was responsible for pre-event planning, estimation of staffing and equipment requirements, engagement with Country Rugby League to identify ambassadors, running the event, and determining the follow-up processes for participants. The working group was viewed favorably by interviewees. One ACCHS Chief Executive Officer (CEO) commented that “getting all the main players (together)... worked really well.” Interviewees expressed positive sentiments about the coordinating agency’s administration and management of the working group, with 1 ACCHS project officer stating she “couldn’t fault this part of the support.” The model of collaboration was seen as a blueprint for a broader range of health promotion activities:

...the staff just came in droves and…having those meetings was just so great. That’s the way we should do all of our health promotion.

ACCHS practice manager

**Table 6:** Interviews by professional category.

Site	Chief executive officer	Clinic manager	General practitioner	Nurse care coordinator	Aboriginal project or liaison officer	Local Hospital District staff	Program staff	Other	Total
1	—^a^	1	—	1	1	—	—	—	3
2	1	—	—	—	1	1	—	—	3
3	—	1	—	1	—	2	—	—	4
4	1	—	—	1	—	2	—	1	5
5	—	—	—	—	—	—	—	—	0
6	—	—	—	1	1	—	—	—	2
7	—	—	1	—	—	—	—	—	1
8	—	—	—	—	—	—	—	—	0
9	—	—	—	1	—	—	—	—	1
Other	—	—	—		—	—	2	—	2
Total	2	2	1	5	3	5	2	1	21

^a^Cells with dashes indicate that no professionals were interviewed in that category.

#### Staff Training

Interviewees considered a lead time of around 3 months was required to adequately plan for an event. In general, pre-event staff training was considered sufficient and was usually conducted as a one-off, half-to-whole-day activity supplemented by training on the event day itself. Staff participation at training events required support from managers and advanced planning to free up staff time for attendance. One interviewee felt it was “up to management pushing that need for people to attend.” At some sites, however, the inability to do pre-event training did not appear to be a major barrier:

no training had been done with the app at all [due to technical difficulties on the training day]... and so we winged it on the day...it took probably about a minute with each person and everyone had the hang of it. So that just proves how simple the app is to use I guess.

LHD coordinator

One manager commented that the training could be more structured and that less experienced staff might benefit from small group learning. Several participants also suggested dedicating more training in the use of the point-of-care machinery.

#### Event Day

#### Implementing a Clinical Program in a Community Setting

Interviewees were generally positive about event day, viewing it as an opportunity for family and friends to come together. Holding events outdoors rather than inside a clinic facility was seen as particularly important:

it’s a good get together...you can’t put money on the value of community getting together... having a yarn and catching up with each other.

ACCHS registered nurse

It was also perceived to be a fun way to learn more about risk factors for chronic disease. One hospital district nurse felt that it went beyond the usual “deficit” model of Aboriginal health and served to empower individuals:

Aboriginal people must get... bored of the statistics thrown at them about chronic disease...and their lifestyle management is causing all of these problems. Whereas I don’t think that’s what 1 Deadly Step did. I think it was a really positive way of getting the message across that you can do something about this and we’re here to help you.

LHD Clinical Nurse Consultant

It also gave staff an opportunity to strengthen relationships with staff from other organizations. These events also have potential to boost ACCHS staff morale and make their work more visible to board members. Some events were held concurrently with a longstanding national cultural event (National Aborigines and Islanders Day Observance Committee, NAIDOC) and this was an effective strategy to *demedicalize* the program:

NAIDOC draws everybody…They don’t really see it as coming here to get screened. They see it as—if I do all these steps I get that cool jersey and I get to have a feed and I get to have a day out with my family.

ACCHS practice manager

However, running an event alongside another community event also increased operational complexity. Aboriginal staff, in particular, have community and family responsibilities at these events in addition to their work responsibilities. This appeared to make management and oversight of the day more difficult. Despite many participants highlighting the importance of holding outdoor events, inclement weather also poses additional challenges such as exposed electrical cords, marquees becoming unstable in the wind, and an increased potential for biological samples to be incorrectly processed, misplaced, or tipped over.

#### Work Flow Considerations

Most people considered the clinical information collected to be important; however, this needed to be balanced against managing the workflow associated with large-scale screening. Consequently, there were mixed views concerning the optimal amount of information that should be collected. Some interviewees considered that all of the data were important as they could support other service activities such as completion of government-rebated Aboriginal health assessments. One ACCHS manager also considered this information of particular importance for improving the quality of their key performance indicator data that is provided to funding bodies. Other interviewees questioned the relative merits of conducting point-of-care testing for cholesterol, diabetes, and kidney disease for all participants (as discussed further).

#### Role of Country Rugby League and Marketing Activities

The use of Country Rugby League ambassadors was considered as a useful community engagement strategy, making the event more fun for children and freeing up parent or carer time for screening. Some sites used the ambassadors to leverage additional marketing opportunities through free local media advertising or via a Facebook page. Although viewed favorably, some interviewees commented that the ambassadors need to be more committed to supporting the program:

there’s a role for ambassadors, but...if...the ambassadors can’t speak passionately about it, people can see straight through that...Often it’s sold as the rugby league player’s going to be there and then they’re not...it’s like a con job.

ACCHS CEO

1 Deadly Step shirts were also critically important incentives to enhance event attendance:

in all the years I’ve been working in Aboriginal health, shirts are a really big incentive, people...love to wear them and they love to promote them.

ACCHS clinic manager

Encouraging the staff to wear the event shirts in the weeks leading to the event was a successful marketing strategy, as patients were asking “How do I get one of the shirts?” Some participants were disappointed that the shirt design remained unchanged from the previous year, further highlighting the importance of refreshing the designs regularly.

#### Technical Challenges

There was general consensus that the iPad app was easy to use and required minimal training*.* The main issue raised by some interviewees was related to the challenges of entering results at the blood test station. Additional problems included an inability to enter an error code when a patient’s result was outside the range of the machine. Some interviewees suggested taking a modular approach to screening events where event organizers could adapt the program to their specific requirements. This included provision of a *light* version of the app that did not include all of the mandatory screening stations.

Several interviewees raised the issue of bottlenecks associated with the blood and urine testing stations. The point-of-care machines take several minutes to process a result, and problems particularly arose when samples had to be rerun because of errors:

...it’s like the brake lights on the highway...Once you start having to...rerun samples and potentially getting the same error…it slows down the flow of people going through.

ACCHS CEO

Some interviewees reported problems in printing patient reports, which was related to the use of older model iPads. Other interviewees commented on problems related to insufficient network capacity and problems with printers. These delays resulted in some participants leaving before receiving their report or having a discussion with GPs and nurses.

#### Postevent

#### Challenges With Follow-Up

Most interviewees considered follow-up activities to be resource intensive. At 1 high-performing site with follow-up rates over 80%, the ACCHS clinic manager reflected that it was around a two-month process to implement adequately:

It was six to eight weeks, and there are still people with low level risk...and we’re still capturing them...But all the ones that we listed as priority, have been followed up.

ACCHS clinic manager

One ACCHS CEO felt that these requirements should be made clearer in the working group when preparing for an event:

...there’s a lot of work in the event, but potentially there’s a heap of work after the event and you need to think through what your follow up strategies are going to be...

ACCHS CEO

Much of the follow-up processes were implemented by managers, administrators, and Aboriginal Health Workers. At sites with staff shortages, these processes were particularly difficult to operationalize. The major challenge was related to participants who nominated a care provider other than the local ACCHS for follow-up. For non-ACCHS participants, it was originally envisaged that the nominated GPs would be registered into the system and notified of the patients who had requested follow-up through them. However, in practice, this process was difficult to implement, and consequently, the coordinating agency modified the process such that a hospital district staff member was given responsibility for follow-up of non-ACCHS participants.

Coordinating follow-up processes between different sectors was seen as a valuable outcome from LHD participation in the program. However, some staff suggested this needed to translate into more tangible benefits to justify their participation. For example, 1 LHD staff member would have liked the follow-up process to go one step further and allow for uploads of patient reports into the hospital record system:

...I suppose the question is what was in it for us?...quite a lot of staff were involved–paid for by the LHD...if we didn't get any access to the results and be able to have some input into those patient's care then why would we be involved in the first place?

LHD care coordinator

One solution to improve follow-up care at some sites was availability of GPs and/or senior nurses on the event day itself for immediate discussion with participants. These sites ensured sufficient privacy for the participant, and it reduced the managerial and administrative staff workload postevent.

### Implementing Population Management Processes

The principal process for follow-up was through the use of the provider-reporting portal. Although not all providers consistently used the portal, those that had used it were generally positive and appreciated its simplicity. In addition to the patient-specific reports, the overall event report summary that was provided to key stakeholders was also generally viewed positively:

I thought it [the event report Summary] was really good...it gives you exactly how many people were screened, how many people nominated the stakeholders as their provider...It highlighted for us as a health provider that you can target programs around some of that data

[ACCHS project officer

Although this report was also reviewed by the working groups, it was unclear to what extent the information was used to inform population health activities. One ACCHS CEO felt that they could benefit from strategic advice on implementation of chronic disease management programs across their community in light of the findings:

it would be useful for somebody to work with the services around what they might do with that information...these are your risk factors in the community...and do some projections around...where it could head.

ACCHS CEO

Several interviewees also commented on the need for greater integration of the data into routine service provision. This included having the ability to upload participant reports directly into the electronic health records and to generate referrals for specific services such as smoking cessation. The current systems allowed only for uploading of static documents into the patient file. There was a strong interest in being able to upload results into the coded fields of the patient record that could then be used to autopopulate items required for key performance indicator reports and for Medicare-rebated health assessments.

### Sustainability

Although stakeholders were generally enthusiastic about involvement in 1 Deadly Step, the trade-off between investing in this program and other activities was raised, particularly by non-ACCHS interviewees:

...they’re really pushing hard for activity-based funding...we certainly had discussions early on about whether it would be possible (to justify)...the time away from people’s usual...work activities.

LHD Clinical Nurse Consultant

The funding provided to sites was generally considered insufficient to cover actual costs, and it appears that a considerable amount of in-kind support (eg, local companies providing free generators) was harnessed to support implementation. Some interviewees suggested that multiple stakeholders should pool resources from existing budgets to rationalize costs:

...we weren’t trying to delegate to each other which can cause controversy with who’s bossing who...We knew exactly what we had to do and, with that, we found we were more inclined to give more back into it.

LHD coordinator

An additional factor that may influence sustainability was the need to establish a system whereby shared learnings from sites could be made available to other sites. One interviewee, for instance, raised the idea that an experienced person from a site that had held a 1 Deadly Step event earlier might mentor those responsible for conducting an event elsewhere.

## Discussion

### Principal Findings

In this paper, we examined multiple data sources to evaluate a community-based chronic disease screening and management program for Aboriginal communities in 1 Australian state. There are three main findings from the evaluation: (1) the clinical profile of participants suggested a high burden of chronic diseases and their risk factors, (2) the program had high satisfaction and acceptability rates with several implementation barriers and enablers identified, and (3) factors that might influence the maintenance and sustainability of the program were observed. These findings have important implications for future iterations of the program.

It is difficult to determine representativeness of the communities in which events were held as people often travel large distances to attend. Therefore, we used a large geographic boundary to determine the draining population and estimated that 6% of the population were screened at these events. When compared with the 2012-2013 Australian Aboriginal and Torres Strait Islander Health Survey (AATSIHS) several observations can be made of the 1 Deadly Step participant profile [14,15]. In terms of lifestyle behaviors, current smoking rates and elevated waist circumference were similar in 1 Deadly Step compared with the AATSIHS. Combined overweight and obesity rates (74% vs 66%), elevated blood pressure (37% vs 20%), dyslipidemia (74% vs 51%), CKD rates (21% vs 17%), and diabetes (21% vs 11%) were all higher in 1 Deadly Step compared with the AATSIHS. Despite being a predominantly nonremote sample, these elevated rates are closer to those observed in people from remote areas in the AATSIHS. The prevalence of self-reported CVD in 1 Deadly Step was similar to those reporting heart disease in the AATSIHS (13% vs 12%). The rates of taking recommended treatments for those with or at high risk of CVD were low (47% for CVD and 36% for high CVD risk) and consistent with previous studies [6,16,17].

There are two important implications from the risk factor and care management information. First, 1 Deadly Step is a useful strategy for identifying people at high risk of chronic disease. To enhance the reach of the program, repeated events are likely to be needed to increase the proportion of eligible community members participating. The risk factor prevalence rates of 1 Deadly Step participants is considerably higher and occurs at younger ages than for non-Indigenous people, and in several areas, these rates are higher than reported in representative surveys of Aboriginal and Torres Strait Islander people. There are clearly substantial opportunities to use the program to address access barriers for this group. Second, substantial gaps in optimal care for those with or at high risk of chronic diseases were observed, and consequently, there are also major opportunities for providing higher quality care for these groups through better linkages with their primary care providers.

Several factors influenced program implementation. Participants were generally highly satisfied with the program, and staff involved in its implementation were positive about their involvement. The ability of 1 Deadly Step to draw on existing *community capital* is perhaps the strongest asset of the program. Implementation of traditional clinical processes into such a setting requires considerable planning and harnessing of resources, and the ability to effectively engage communities in this process is noteworthy. The use of existing community events such as NAIDOC, judicious marketing through local and social media, involvement of country rugby league ambassadors, and coordination of activities via a local working group were highly effective strategies to support program participation. Staff from all health service sectors and levels within their organizations demonstrated immense enthusiasm for conducting activities that could address the high–chronic disease burden experienced by Aboriginal communities. Staff welcomed the opportunity to work collaboratively and showcase their efforts to the community. The coordinating agency played a critical role in supporting implementation of the program. Given the resource constraints under which these staff were working, this level of support for the program was an essential enabler to its successful implementation. The ability to be locally responsive while at the same time providing a *macrolevel* view of the program is an important success factor.

A number of areas were also identified where the program was hindered by specific challenges. The most substantial issue was related to the follow-up of participants, particularly those who nominated another service provider other than the local ACCHS. Follow-up rates were highly variable across sites and suggest that there are particular local service issues at play. This has been documented in previous research conducted in the Northern Territory and Queensland [18]. Services with high follow-up rates appear to have committed substantial internal resources to supporting follow-up. Some sites were able to effectively integrate 1 Deadly Step into existing operational processes and used it as an opportunity to strengthen their reporting capacity to funders. This highlights the challenge of intersector collaboration between local hospital districts, private GPs, ACCHSs, and other agencies, given that there are different jurisdictional responsibilities, information systems, and care processes across these stakeholder groups.

There were also a range of technical hurdles that needed to be overcome. These appeared related to the use of point-of-care machines, network connectivity, and entering data on the iPad software itself. Aside from fixing technical bugs, a major implication is determining the most feasible amount of clinical information that can be collected. Some software modifications could be considered to reduce the amount of information collected at an event. A modular approach could be taken to screening where only certain high-risk subgroups need to have blood and urine testing. A “low-information” algorithm for risk prediction could also be used that was less reliant on complete risk factor information, and this could considerably shorten screening time and reduce bottlenecks. Such algorithms have been developed and validated in overseas populations but would likely need some adaptation and validation work before being used in this setting [19]. Balanced against this, however, is that high–risk factor prevalence rates observed in this program were apparent for a large proportion of the population screened. This provides a strong justification for comprehensive screening. Thus, there is an inherent tension between collecting sufficient information to make the clinical assessment meaningful and overburdening services and participants on the event day.

There are several opportunities that could be derived from 1 Deadly Step. The most important is to integrate 1 Deadly Step data into service processes, thus making it a part of routine business planning and operations [20]. Integration with electronic health record systems would allow providers easy access to screening assessment data in much the same way as specialist and pathology reports are currently viewed, actioned by staff, and extracted for auditing and recall processes [21]. This would also enhance reporting requirements to funders; increase capacity to meet key performance indicator requirements; drive referral to other services; and generate business revenue opportunities, such as meeting the requirements for government-rebated preventive health assessments [22]. Another important opportunity to build on the strengths of the 1 Deadly Step program is to foster collaboration and learning between communities that have participated in events. Currently, these events are run in a stand-alone manner; however, it would be worth considering the establishment of a *learning collaborative* in which nominated representatives could share resources, determine optimal operational approaches, benchmark their performance, and develop data-driven strategies to address follow-up challenges [23]. As consumer-controlled electronic health records become more prominent, integration of screening assessments into these systems could also enhance engagement and promote better exchange of information between care providers [24].

### Limitations

This evaluation comes with some important limitations. The sample attending 1 Deadly Step events was a relatively small convenience sample, and therefore, it may not be representative of the local communities participating. The 9 events were from a variety of locations across rural and urban settings in 1 Australian state; therefore, they may not be generalizable to other parts of Australia. The satisfaction survey response rate was low and could be prone to respondent bias. The assessment of the use of guideline-recommended medications was based on self-reported data that is subject to recall bias [25]. The information on follow-up visits was based on reporting activity in the Web portal. Although all health services were regularly encouraged to keep this information up to date, there may be some attendees who received follow-up care, but this was not recorded in the portal.

Taking into consideration some of the existing strengths of the current program, the major challenges faced by 1 Deadly Step relate to its ability to sustain current levels of activity and scale-up activity across NSW. Considerable effort was expended to run these 9 events, and there is a risk that the goodwill associated with this may diminish, particularly as competing demands may displace 1 Deadly Step for other higher-priority areas. Therefore, it is important that the value proposition to staff and stakeholder organizations be high [26]. Real program costs need to be assessed to allow stakeholder organizations to gain a better understanding of the resource requirements to implement 1 Deadly Step. In parallel, it is also important to assess any downstream impact the program may have in terms of savings to the health system made through earlier intervention for chronic disease risk factors and management. An economic evaluation that links 1 Deadly Step participants to other routinely available datasets may aid in addressing this question.

### Conclusions

1 Deadly Step was implemented in 9 communities in 2015-2016 and assessed the chronic disease risks for over 1000 Aboriginal people residing in these communities. The clinical data strongly support the justification for such a program given the high levels of risk factors encountered, often including people who would otherwise have had no knowledge of these issues before the events. Overall, the event implementation was highly successful and demonstrated high satisfaction by participants and staff alike. However, several challenges were highlighted, particularly in relation to resource constraints and follow-up processes. Several opportunities were identified to address these issues, and these are likely to play a critical role in influencing program sustainability. It is important to note that despite the successful implementation of the program, its effects in improving health outcomes remain unknown and a more detailed impact evaluation with long-term follow-up is needed to assess downstream system and health benefits.

## Appendix

Multimedia Appendix 1 Samples of the screening application.

Multimedia Appendix 2 Administrator portal.

Multimedia Appendix 3 Provider portal.

Multimedia Appendix 4 Logic model.

## Abbreviations

AATSIHS: Australian Aboriginal and Torres Strait Islander Health Survey
ACCHS: Aboriginal Community Controlled Health Service
BMI: body mass index
CEO: chief executive officer
CKD: chronic kidney disease
CVD: cardiovascular disease
GP: general practitioner
HbA_1c_: glycated hemoglobin
LHD: local hospital district
NAIDOC: National Aborigines and Islanders Day Observance Committee
NSW: New South Wales
