# Supplementary figures and images for: Community-Based Chronic Disease Prevention and Management for Aboriginal People in New South Wales, Australia: Mixed Methods Evaluation of the 1 Deadly Step Program
Source: JMIR Mhealth Uhealth. 2019 Oct 21;7(10):e14259. doi: 10.2196/14259 (PMC6913719; doi:10.2196/14259)

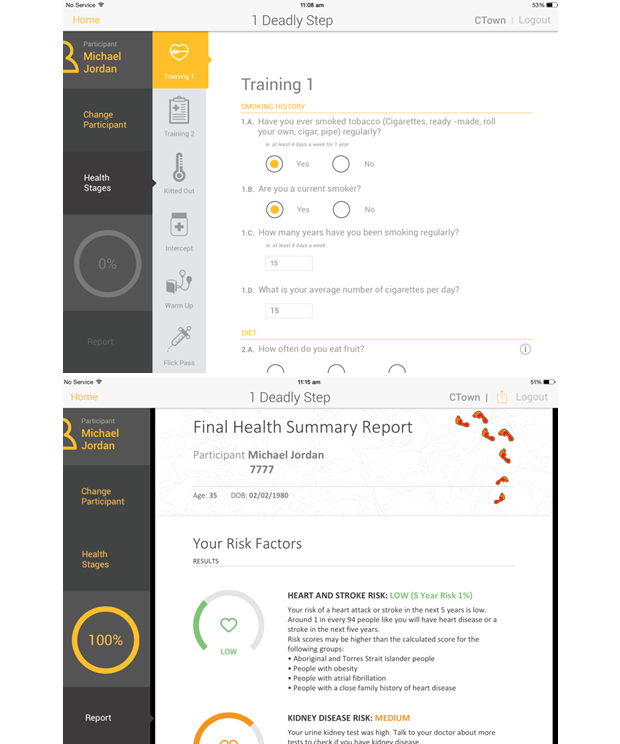

Supplement: Multimedia Appendix 1 [file 1deadlystepSup1.png]

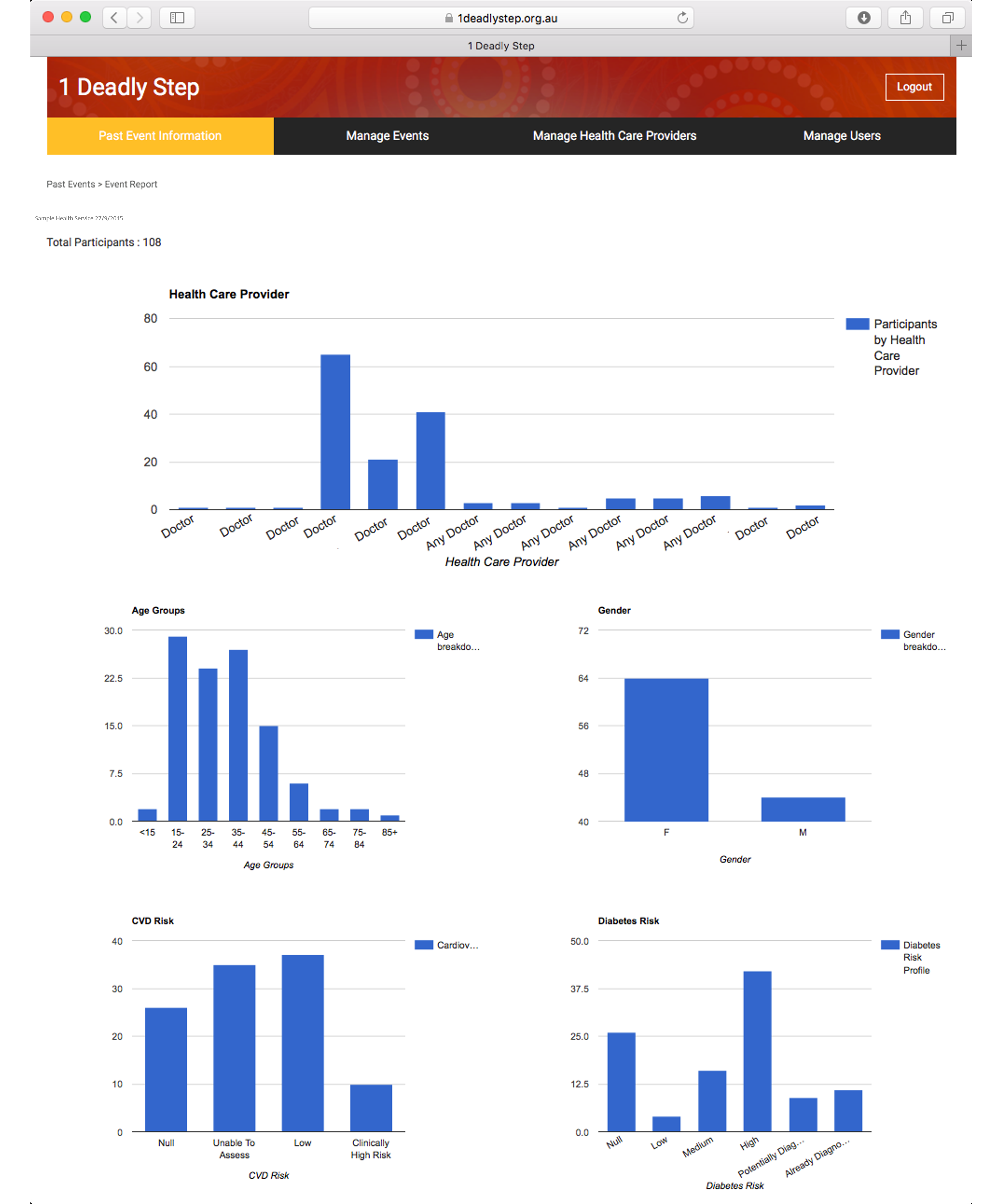

Supplement: Multimedia Appendix 2 [file 1deadlystepSup2.png]

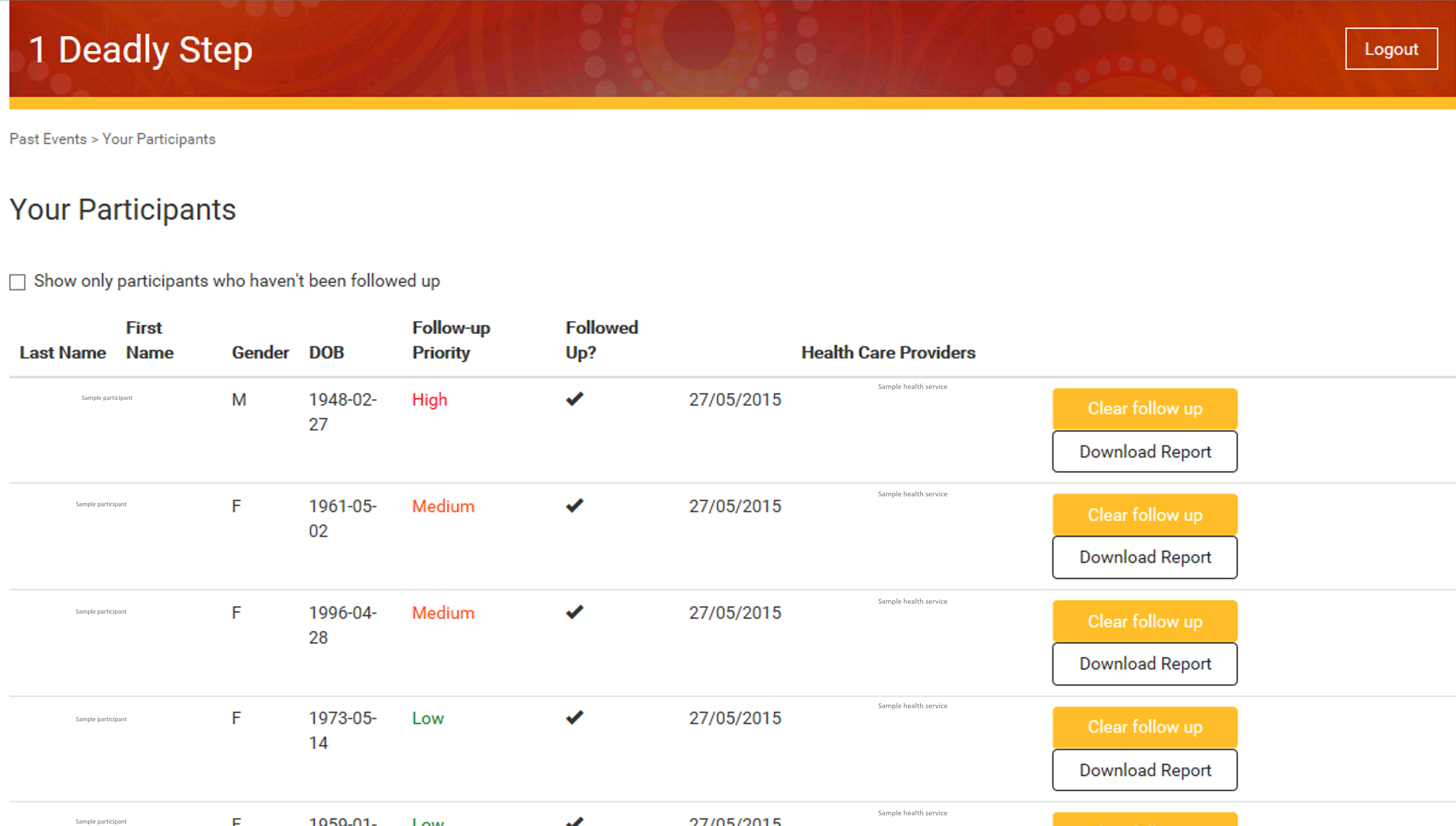

Supplement: Multimedia Appendix 3 [file 1deadlystepSup3.png]

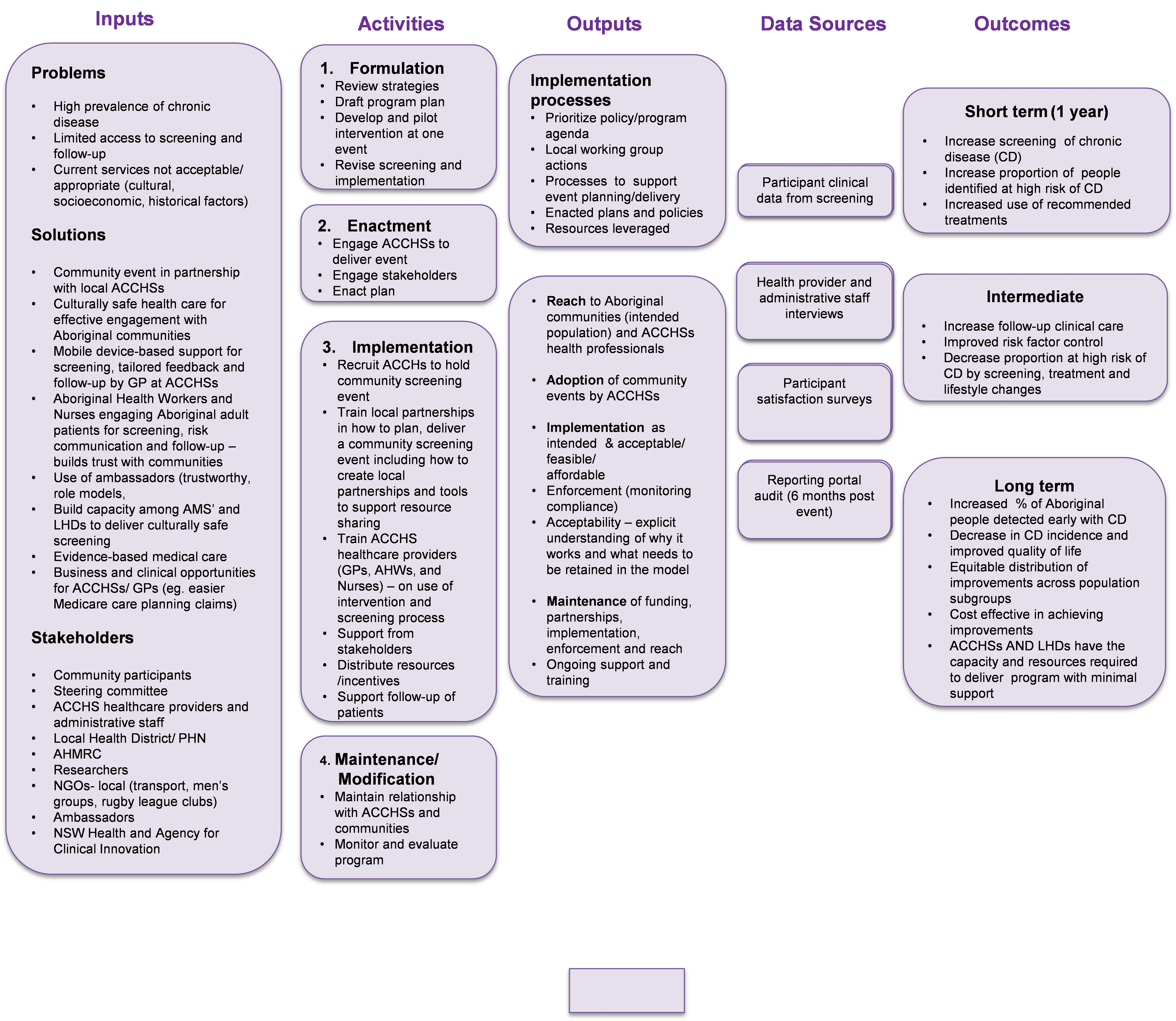

Supplement: Multimedia Appendix 4 [file 1deadlystepSup4.png]
